# Quantification and Purification of Mangiferin from Chinese Mango (*Mangifera indica* L.) Cultivars and Its Protective Effect on Human Umbilical Vein Endothelial Cells under H_2_O_2_-induced Stress

**DOI:** 10.3390/ijms130911260

**Published:** 2012-09-10

**Authors:** Fenglei Luo, Qiang Lv, Yuqin Zhao, Guibing Hu, Guodi Huang, Jiukai Zhang, Chongde Sun, Xian Li, Kunsong Chen

**Affiliations:** 1 Laboratory of Fruit Quality Biology/The State Agriculture Ministry Laboratory of Horticultural Plant Growth, Development and Quality Improvement, Zhejiang University, Zijingang Campus, Hangzhou 310058, China; E-Mails: fenglei2012@zju.edu.cn (F.L.); lvqiang_85126@126.com (Q.L.); zhjk2010@zju.edu.cn (J.Z.); adesun2006@zju.edu.cn (C.S.); akun@zju.edu.cn (K.C.); 2 College of Pharmaceutical Sciences, Zhejiang University, Zijingang Campus, Hangzhou 310058, China; E-Mail: zhaoy@hotmail.com; 3 College of Horticulture, South China Agricultural University, Guangzhou 510642, Guangdong, China; E-Mail: guibing@scau.edu.cn; 4 Guangxi Subtropical Crop Research Institute, Nanning 530001, Guangxi, China; E-Mail: gxhgd@126.com

**Keywords:** *Mangifera indica*, mangiferin, purification, antioxidant, HUVEC, protective effect

## Abstract

Mangiferin is a natural xanthonoid with various biological activities. Quantification of mangiferin in fruit peel, pulp, and seed kernel was carried out in 11 Chinese mango (*Mangifera indica* L.) cultivars. The highest mangiferin content was found in the peel of Lvpimang (LPM) fruit (7.49 mg/g DW). Efficient purification of mangiferin from mango fruit peel was then established for the first time by combination of macroporous HPD100 resin chromatography with optimized high-speed counter-current chromatography (HSCCC). Purified mangiferin was identified by both HPLC and LC-MS, and it showed higher DPPH^•^ free-radical scavenging capacities and ferric reducing ability of plasma (FRAP) than by l-ascorbic acid (Vc) or Trolox. In addition, it showed significant protective effects on human umbilical vein endothelial cells (HUVEC) under H_2_O_2_-induced stress. Cells treated with mangiferin resulted in significant enhanced cell survival under of H_2_O_2_ stress. Therefore, mangiferin from mango fruit provides a promising perspective for the prevention of oxidative stress-associated diseases.

## 1. Introduction

Mango (*Mangifera indica* L.), the most important fruit in *Anacardiaceae* family, is a tropical fruit with high nutritional and medicinal value. It originated from the Asia Indo-Burmese region approximately 4000 years ago, and is now commercially grown in more than 87 countries [1]. Morphologically, mango fruit belongs to drupe, where the pericarp is distinguished into smooth exocarp (peel), fleshy mesocarp (pulp), and stony endocarp (kernel). Fully ripe mango is famous for its strong aroma, intense peel coloration, delicious taste, high amount of bioactive compounds such as phenolic compounds, β-carotene, vitamin C, and minerals. The chemical composition of mango varies with the location of cultivation, variety, stage of maturity, *etc*. [2–5].

**Figure 1:**
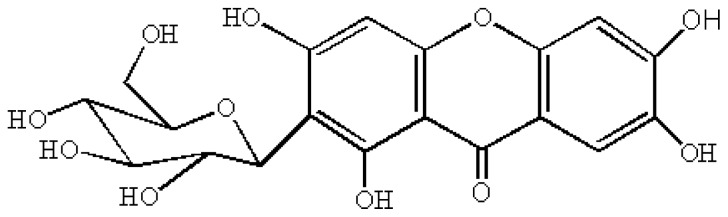
Structure of mangiferin.

Among the various polyphenolic compounds found in the mango [6], mangiferin (C-2-β-d-glucopyranosyl-1,3,6,7-tetrahydroxyxanthone, also named C-glucosyl xanthone) is a distinct one (Figure 1). Mangiferin is a heat-stable molecule and a natural pharmacologically active phytochemical that was found to have various bioactivities, such as anti-inflammation [7], anti-diabetic [8], immunomodulatory [9], anti-tumor [10,11], and antioxidant [12,13]. It can promote endothelial cell migration during the angiogenesis and may therefore have promising prevention and therapeutic potentials on vascular diseases [14]. It bears a catechol moiety, which is important for its diverse biological activity.

Due to its potential bioactivity, there are many studies researching the distribution of mangiferin in the plant kingdom. So far, 12 families have been recorded, with *Anacardiaceae* and *Anemarrhena* being two main resources [6]. As an important secondary metabolite to protect the plants against various forms of biotic and abiotic stress, higher amounts of mangiferin were detected in the leaves, barks, fruit peel of *Mangifera indica* Linn than the edible mango pulp [13,15,16].

As the second largest producer of mango fruit in the world, China (4.14 million MT, 2008 FAO data) has a long history of mango cultivation and there are currently approximately more than 300 mango cultivars. However, research into the characterization of bioactive compounds in mango cultivars in China is quite rare. Recently, Ma *et al*. [3] reported for the first time on the polyphenolics compounds and antioxidant activities in mango cultivars in China. However, no extensive work has been carried out to investigate mangiferin distribution in Chinese mango fruit. The present study was designed to quantify the mangiferin content of 11 Chinese mango cultivars and to isolate mangiferin from mango peel, using a combination of macroporous resin chromatography and HSCCC for evaluation of its antioxidant activity and protective effect on human umbilical vein endothelial cells (HUVEC) under H_2_O_2_-induced stress.

## 2. Results and Discussion

### 2.1. Quantification of Mangiferin in Different Parts of 11 Mango Cultivars

By dissecting mango fruit into three parts, namely pulp, peel, and seed kernel, mangiferin content in both edible and non-edible parts of the mango fruit was characterized by 11 Chinese cultivars in the present study. Significant difference in mangiferin content among different cultivars as well as different tissues of mango fruit was shown in Table 1. High mangiferin contents were found in the peel of mango cultivars such as LPM (7.49 mg/g DW), ZHM (7.34 mg/g DW), and seed kernel of ZHM (2.43 mg/g DW), XH-2 (1.04 mg/g DW), *etc.* Therefore, mango peel and seed kernel are good sources of mangiferin.

Mangiferin content was only detected in the flesh of five Chinese mango cultivars tested, with the highest content of 0.20 mg/g DW. However, due to its high biological and medicinal activity, mangiferin content in the edible fruit tissues still attracts the attentions of many researchers and consumers. Mangiferin in commercial mango puree concentrate (MPC) was found to reach a level of about 4.4 mg/kg MPC based on HPLC analysis and mass spectrometric detection [17]. Mangiferin in the flesh four Brazilian mango varieties range from undetectable to 12.4 mg/kg DW (Ubá variety) [5]. Recently, a high mangiferin level (227.4–996.1 μg/g FW mango puree) was found in Mexico Ataulfo cultivars among five cultivars tested, and more mangiferin accumulated in late-harvested mango than the early-harvested ones within the same cultivar in the same year [4].

### 2.2. Purification of Mangiferin from LPM Peel

So far, few efficient separation methods have been reported for purification of this natural bioactive compound from mango, which prevented its further characterization and comprehensive utilization. Preparative isolation of mangiferin was reported from the well-known traditional Chinese medicinal herb *Anemarrhena asphodeloides* [18]. However, the preparation method reported for *Anemarrhenae* cannot be directly and completely applicable to *Mangifera indica* due to the diversity and complexity of the plant’s secondary metabolites in different plant materials.

**Figure 2:**
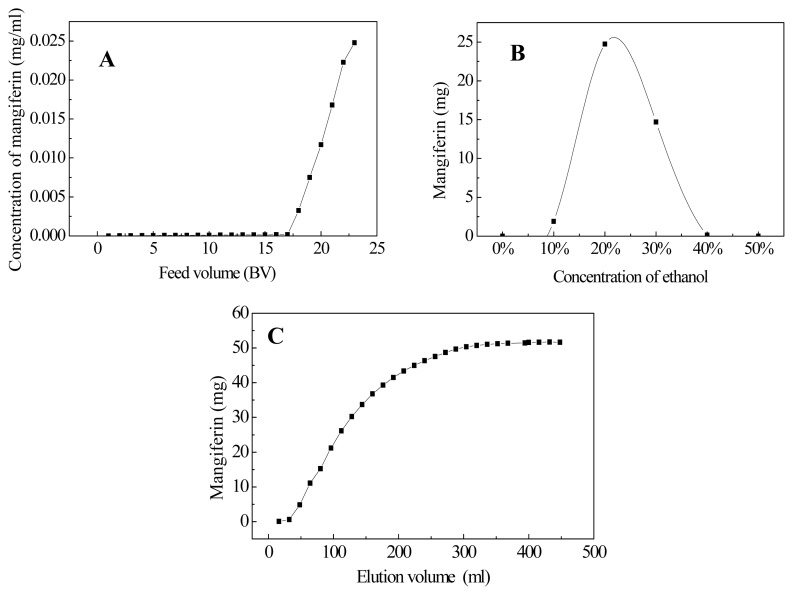
Dynamic leakage curves (**A**), gradient elution curve (**B**), and isocratic desorption curve (**C**) of mangiferin on column packed with HPD100 resin. Initial mangiferin concentration in feed solution: 0.25 mg/mL; isocratic desorption solution: 30% *v*/*v* ethanol; both adsorption and desorption flow rate: 2 mL/min.

Based on our preliminary experiment, HPD100 showed good adsorption capacity towards mangiferin. Dynamic adsorption and desorption experiments showed that the breakthrough volume of mangiferin on HPD100 resin was about 17 BV (Figure 2A), and the majority of mangiferin absorbed by HPD100 resin was eluted by 20%–30% ethanol solutions (Figure 2B). Therefore, 30% ethanol aqueous solution was used as the eluent in isocratic elution, where complete desorption was done with an elution volume of about 320 mL (8 BV) (Figure 2C). All the eluents were collected and the one-step macroporous resin chromatography efficiently enriched mangiferin in the *Mangifera indica* sample, which was used for further HSCCC purification.

A successful HSCCC separation of desired compound depends largely upon the selection of a suitable two-phase solvent system, which provides an ideal *K* value at around 0.5–2. In the present study, seven solvent systems were tested for the selection of ideal *K* value for mangiferin (Table 2). Results showed that the *K* values from *n*-butanol–1% acetic acid (1:1, *v*/*v*), *n*-butanol–methyl alcohol–1% acetic acid (1:0.1:1, 1:0.2:1) were too large, which meant mangiferin was mainly dissolved in the upper-phase and was hence not suitable for better separation. In contrast, the *K* values from ethyl acetate–water (1:1, *v*/*v*), ethyl acetate–methyl alcohol–water (1:0.1:1, 1:0.3:1) were too small, which meant mangiferin was mainly dissolved in the lower-phase and was also unsuitable for better separation. The ethyl acetate–*n*-butanol–water (4:1:5) solvent system showed ideal *K* value of 1.76 for mangiferin, which resulted in good separation of mangiferin from the resin-refined sample.

**Figure 3:**
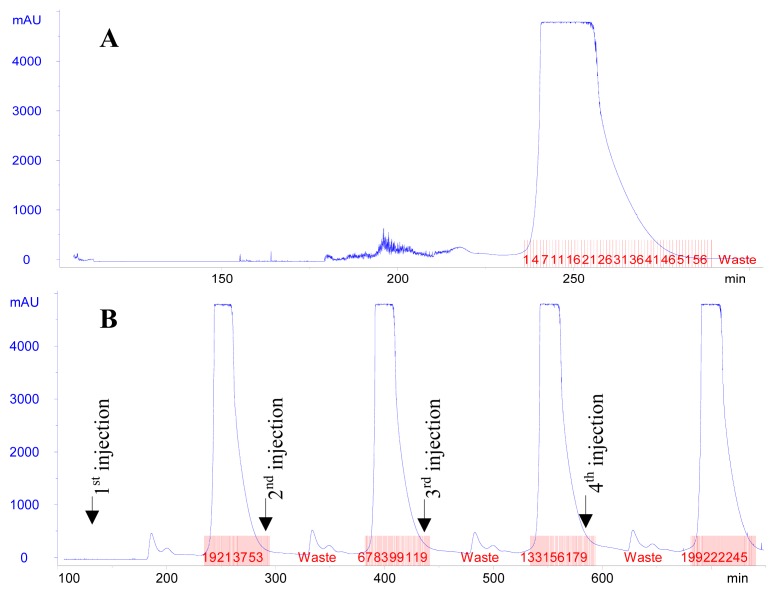
High-speed counter-current chromatography (HSCCC) chromatogram of separation of mangiferin from resin-refined sample (**A**: Single injection mode; **B**: Continuous injection mode). Two-phase solvent system: ethyl acetate–*n*-butanol–water (4:1:5, *v*/*v*/*v*); stationary phase: upper phase; mobile phase: lower phase; flow rate: 2.0 mL/min; revolution speed: 800 rpm; detection wavelength: 258 nm.

For single injection mode, effluents in tubes from #6 to #46 were proved by HPLC analysis to contain pure mangiferin and they were hence combined (Figure 3A). For continuous injection mode, effluents in tube #10-50, tube #77-117, tube #142-182, and tube #209-249 were proved to contain pure mangiferin and they were hence combined (Figure 3B).

**Figure 4:**
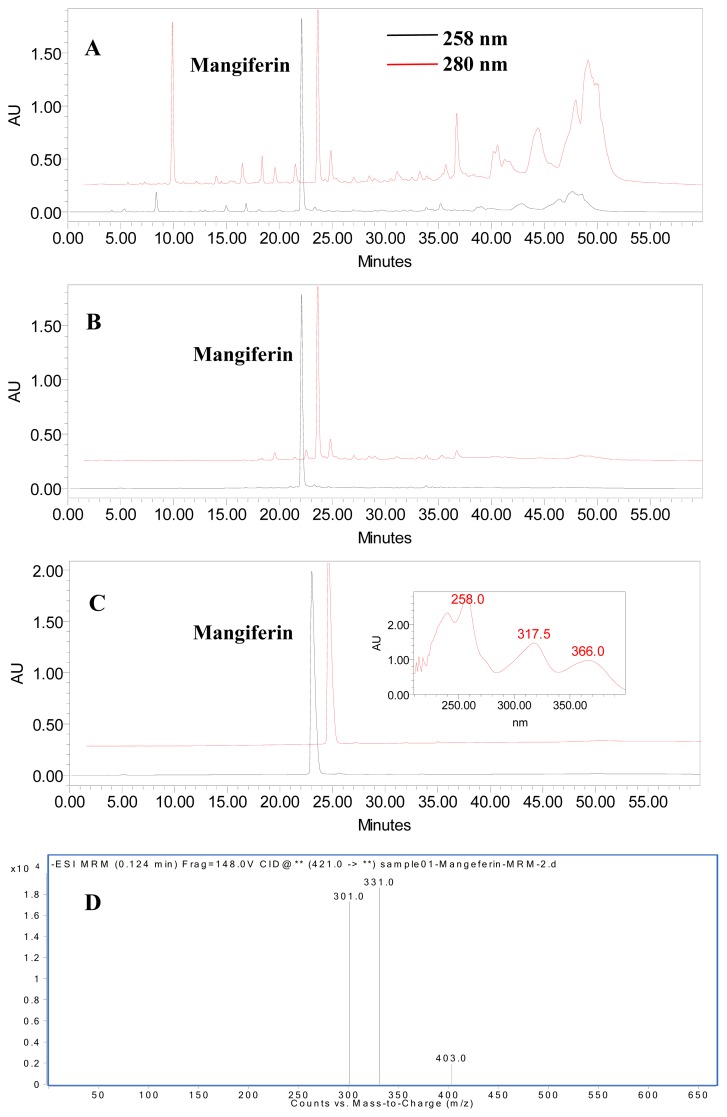
High performance liquid chromatography (HPLC) chromatogram of crude extract before (**A**) and after (**B**) treatment with HPD100 resin, the HSCCC purified product (**C**), and LC-MS chromatogram of the final purified mangiferin (**D**) (*λ*= 258 nm).

The effect of the two-step purification was shown in Figure 4, and the purities and recoveries of mangiferin in different procedures were summarized in Table 3. The purity of mangiferin in the crude extract of LPM peel was as low as 0.75%. According to the HPLC chromatograms, the majority of UV absorbing impurities were removed by HPD 100 resin chromatography (Figure 4A,B). After one-step HPD100 resin purification, the purity increased to 37.80%, which was 50.4-fold that of the crude extract. The mangiferin recovery was 85.15% (Table 3). In addition, HSCCC purification resulted in 74.3 mg mangiferin with 99.13% purity (Figure 4C) and the recovery rate was 77.94% from a 250 mg sample (Table 3). Further identification of the purified mangiferin was confirmed by HPLC-ESI-MS (Figure 4D), where the [M-H]^−^ ion at *m*/*z* 421.0 indicating the molecular weight of mangiferin to be 422. Additionally, three C-glycoside xanthone typical product ions at *m*/*z* 403.0 [M-H_2_O]^−^, 331.0 [M-C_3_H_6_O_3_]^−^, 301.0 [M-C_4_H_8_O_4_]^−^ were detected in LC-MS_2_ chromatogram, the fragmentation pattern of which is consistent with the proposed mechanistic pathway by Ma *et al.* [19]. Based on these data, the structure of the purified compound could be identified as mangiferin (C_19_H_18_O_11_). Therefore, both the HPLC and LC-MS data confirmed the purified compound to be mangiferin. These results demonstrated that HPD100 resin chromatography combined with optimized HSCCC resulted in efficient purification of mangiferin from LPM peel.

Due to the limited time of the harvest season, mango fruit is consumed both freshly and as a processed production, the latter of which can be mango juice, mango nectar, canned mango slices, mango jam, mango beverages, mango puree, mango powder, *etc.* The byproducts of mango processing are waste in the fruit industry. Therefore, the established mangiferin purification method is better for further comprehensive utilization of mango processing byproducts.

### 2.3. Antioxidant Capacity of Purified Mangiferin

**Figure 5:**
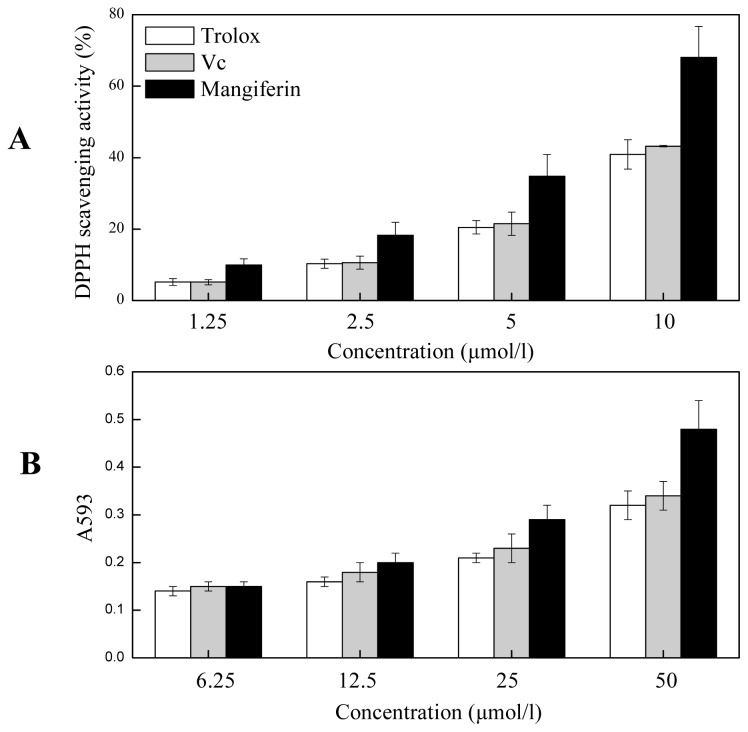
Antioxidant activity of mangiferin assayed by 2,2-diphenyl-1-picrylhydrazyl (DPPH) (**A**) and ferric reducing ability of plasma (FRAP) (**B**).

Oxidative stress plays an important role in the pathogenesis of many chronic diseases such as atherosclerosis and cardiovascular disease and it is well established that dietary polyphenolics compounds play significant roles in the prevention of these stress-associated diseases [20]. In the present study, the purified mangiferin was analyzed for their DPPH^•^ scavenging activity and reducing power by FRAP. At all the concentrations tested, mangiferin showed higher radical scavenging capacity than that of l-ascorbic acid (Vc) or Trolox, and it showed dose-responsive effect in both antioxidant assays. It showed 68.03 ± 0.21% DPPH^•^ scavenging rate at the concentration of 10 μmol/L (Figure 5A). In FRAP assay, the reducing power enhanced with the increased concentrations of mangiferin and it was significantly higher than that of Vc or Trolox at the concentration of 50 μmol/L (Figure 5B).

### 2.4. Protective Effect of Mangiferin on HUVEC under H_2_O_2_-induced Stress

**Figure 6:**
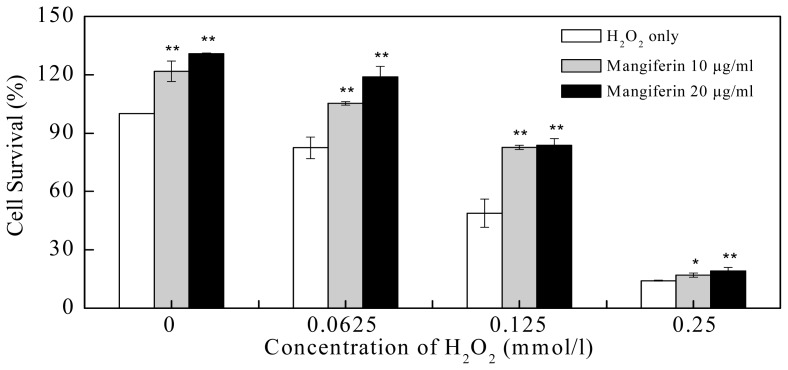
Protective effect of purified mangiferin on human umbilical vein endothelial cells (HUVEC) under H_2_O_2_-induced stress for 24 h. The rates of cell survival are shown as the mean ± SD (*n* = 3). * *p* < 0.05, ** *p* < 0.01, compared to H_2_O_2_ treatment only (as each control) respectively.

Intracellular balance of oxidant and antioxidant mechanisms determines endothelial function. Increasing oxidative stress can cause significant cell toxicity or even apoptosis, impair endothelial functions, and may increase the risk of chronic vascular disease such as atherosclerosis, cardiovascular injury, diabetic angiopathies, *etc.* [21,22]. In the present study, by using the model system HUVEC, the survival rates of cells exposed to varying concentrations of H_2_O_2_ with or without exogenous antioxidant mangiferin were evaluated. Results showed that H_2_O_2_ treatment of HUVEC caused significant decrease of cell survival. Compared with the control (0 mmol/L of H_2_O_2_), the cell survival rates were only 82.45 ± 5.47%, 48.87 ± 7.27%, and 14.14 ± 0.25% after 0.0625, 0.125, or 0.25 mmol/L of H_2_O_2_ treatment, respectively (Figure 6). Mangiferin at concentration of 10 μg/mL showed significant protective effect on HUVEC with the survival rates of 105.29 ± 0.93% under 0.0625 mmol/L of H_2_O_2_ (*p* < 0.01), 82.70 ± 1.08% under 0.125 mmol/L of H_2_O_2_ treatment (*p* < 0.01), 17.00 ± 1.08% under 0.25 mmol/L of H_2_O_2_ treatment (*p* < 0.05), when compared with their own controls (H_2_O_2_ treatment only), respectively. At a concentration of 20 μg/mL, mangiferin treatment resulted in the survival rates of 118.89 ± 5.49%, 83.83 ± 3.30%, and 19.23 ± 1.67% under 0.0625, 0.125, or 0.25 mmol/L of H_2_O_2_ treatment, respectively, which were all significant higher than their own controls (*p* < 0.01) (Figure 6).

The protective effect of mangiferin on HUVEC against glycated protein-iron chelate induced toxicity were previously associated with antioxidant enzymes such as catalase, superoxide dismutase (SOD), glutathione peroxidase (Gpx), and glutathione reductase (GR) [23]. By using the flavonoid-rich apple extract, the down-regulation of oxidant-responsive transcription factor NF-kappaB signaling was proposed as the indicative of the antioxidant effect of apple flavonoids in HUVEC [24]. The anti-inflammatory properties observed for ellagic acid in IL-1β-treated HUVEC was proposed to be the result of inhibition on vascular cell adhesion molecule-1 (VCAM-1) expression and NF-kappaB activation, which may be caused by the ROS radical scavenging activities of ellagic acid [25]. In the present study, the purified mangiferin from mango showed protective effect on H_2_O_2_-treated HUVEC in a dose-responsive manner and its mechanism remains further investigation.

## 3. Experimental Section

### 3.1. Chemicals

The authentic mangiferin was bought from Sigma-Aldrich (St. Louis, MO, USA). It is a white to light-yellow powder with purity of more than 98% (conforms to proton NMR spectra). 2.2-diphenyl-1-picrylhydrazyl (DPPH^•^), l-ascorbic acid (Vc), (±)-6-hydroxy-2,5,7,8-tetramethylchromane- 2-carboxylic acid (Trolox), 2,4,6-tris(2-pyridyl)-*s*-triazine (TPTZ), SRB, and acetonitrile of chromatographic grade were all purchased from Sigma-Aldrich. HPD100 macroporous resin was bought from Tianjin Bohong Resin Technology Co., Ltd. HG-DMEM medium was obtained from GIBCO (Grand Island, NY), and fetal bovine serum (FBS) was from Hangzhou Sijiqing Biotec Co. (Zhejiang, China). Double-distilled water (ddH_2_O) was used in all experiments and samples for HPLC and LC-MS were filtered through 0.22 μm membrane before injection. All the other reagents of analytical grade were bought from Sinopharm Chemical Reagent Co., Ltd. (Shanghai, China).

### 3.2. Materials

Eleven cultivars of mango (*Mangifera indica* L.) fruit used in this study, including Guifei (GF), Guire-265 (GR-265), Jinhuangmang (JHM), Lvpimang (LPM), Liuxiangmang (LXM), Maqiesu (MQS), Sililanka-811 (SLLK-811), Tainong-1 (TN-1), Xinhong-2 (XH-2), Yuexi-1 (YX-1), and Zihuamang (ZHM) were harvested from the Germplasm Collection of Guangxi Subtropical Crops Research Institute, Nanning, Guangxi, China, and all the fruits were allowed to ripen at room temperature until fully ripen according to firmness (around 2–4 N without peel, measured by TA-XT2i texture analyzer fitted with a 5 mm diameter probe, Stable Micro Systems (Godalming, Surrey, UK)), color, and formation of aroma of mango characteristics. Fifteen fruit per cultivar, five for each of three replicates, was manually separated into peel, pulp, and seed kernel, and cut into small pieces before frozen in liquid nitrogen. After freeze-drying (FM 25EL-85, VirTis (Gardiner, NY, USA)), they were ground into fine powder and stored at −80 °C until analysis. HUVEC was cultured and stored in liquid nitrogen in the College of Pharmaceutical Sciences, Zhejiang University before use.

### 3.3. HPLC Analysis of Mangiferin

Mangiferin was extracted from mango fruit samples according to the method described by Noratto *et al.* [10] with some modifications. The ground powder of different fruit tissues (0.5 g) was first extracted with 10 mL of a solvent mixture (ethanol–methanol–acetone, 1:1:1) for 2 h followed by sonication in an ultrasonic bath for 30 min, and the solution was then centrifuged at 10,000 rpm for 10 min at ambient temperature. The sample was extracted twice and the supernatants were combined and used for mangiferin content analysis.

Mangiferin was analyzed by HPLC according to the previous publication with some modifications [26]. Briefly, the HPLC system (2695 pump, 2996 diode array detector, Waters, Milford, MA, USA) coupled with an ODS C18 analytical column (4.6 × 250 mm) was used with the detection wavelength of 258 nm. The mobile phase of HPLC consisted of 2% acetic acid (A) and acetonitrile: 0.5% acetic acid (1:1, *v*/*v*) (B). The gradient elution was performed as follows: 0–2 min, 5% of B; 2–10 min, 5%–25% of B; 10–40 min, 25%–55% of B; 40–45 min, 55%–90% of B; 45–50 min, 90%–55% of B; 50–55 min, 55%–5% of B; 55–60 min, 5% of B. The flow rate was 0.6 mL/min, the injection volume was 10 μL, and the column temperature was 25 °C. Mangiferin was identified according to retention time and peak purity was evaluated using the DAD spectrum. Mangiferin was then quantified based on peak area and comparison with the standard curve. In order to plot the mangiferin standard curve, a serial dilution was made on the mangiferin stock solution (1.0 mg/mL) with 80% methanol to prepare standard solutions at concentrations of 0.1, 0.2, 0.4, 0.6, 0.8, and 1.0 mg/mL.

### 3.4. Purification of Mangiferin from Mango Fruit

The ground powder of LPM peel (20 g) was first treated with 400 mL of light petroleum for 2 h followed by sonication in an ultrasonic bath for 30 min. After removing the lipophilic impurities by light petroleum, the sample was extracted twice with 400 mL extraction solution (ethanol–methanol–acetone, 1:1:1, *v*/*v*) in the ultrasonic bath for 30 min each time. Both extracts were combined and evaporated to dryness at 45 °C. The extract was dissolved in 400 mL of ddH_2_O and extracted twice with 400 mL ethyl acetate to remove impurities with weak polarity.

#### 3.4.1. Macroporous Resin Purification

Purification of mangiferin was carried out by combination of macroporous resin chromatography with HSCCC according to Zhou *et al*. [18] with modification. For macroporous resin chromatography, the lower phase of the ethyl acetate extract was loaded on HPD100 resin column (16 × 400 mm) with bed volume (BV) of 40 mL. The initial feed concentration was 0.25 mg/mL mangiferin and the flow rate was 2 mL/min. Dynamic adsorption and desorption experiments were first carried out to determine the loading volume and the elution conditions. The column was washed thoroughly by ddH_2_O and 5% ethanol to remove the impurities. Gradient elution of mangiferin from HPD100 column was carried out by increased concentrations of ethanol (10%, 20%, 30%, 40%, and 50%) successively. All the effluent was collected for HPLC analysis and samples with enriched mangiferin were evaporated at 45 °C under vacuum for further HSCCC purification.

#### 3.4.2. HSCCC Purification

HSCCC (TBE-300A, Shanghai Tauto Biotech. Co., Ltd, Shanghai, China) in combination with ÄKTA FPLC™ (GE Healthcare Life Sciences, Piscataway, NJ, USA) was used to further purify mangiferin. The HSCCC was equipped with three polytetrafluoroethylene (PTFE) coil separation columns (diameter of tube, 1.6 mm; total volume, 260 mL) and a 20-mL sample loop. The temperature of the HSCCC column was controlled at 25 °C by HX1050 constant temperature circulating waterbath (Boyikang Lab Instrument, Beijing, China). Data were collected by a Unicorn 5.11 chromatography workstation. The two-phase solvent system was selected according to the partition coefficient (*K* value) of mangiferin. First, suitable amount of resin-refined sample was dissolved in different pre-equilibrated solvent systems (upper phase/lower phase, 1:1, *v*/*v*) and vortexed for mixture (Table 2). After equilibration, both the upper phase and the lower phase were analyzed by HPLC. The *K* value of mangiferin in different solvent systems was determined by measuring the peak area in the upper phase and lower phase, recording as *A*_1_ and *A*_2_, respectively, *K* value = *A*_1_/*A*_2_.

The selected solvent of 4000 mL was added into a separating funnel and equilibrated for the formation of the two phases. The upper and lower phases were then separated and degassed by sonication for 30 min. The column of HSCCC was firstly filled with the upper phase solvent at a flow rate of 20 mL/min. The apparatus was rotated at 800 rpm and the lower phase was pumped into the column at a flow rate of 2 mL/min. After hydrodynamic equilibrium was reached, stationary phase retention was calculated as 35%. For single injection mode, after hydrodynamic equilibrium, 50 mg resin-refined sample powder dissolved in 5 mL lower phase solvent was injected. For continuous injection mode, 50 mg sample was injected every 2.5 h after the previous injection. The effluent of the column was monitored by a UV detector at 258 nm and each tube (1.8 mL/tube) was collected for HPLC analysis. Tubes with pure mangiferin were combined for further analysis.

### 3.5. LC-MS Analysis

Further LC-MS analysis was performed using Agilent 6430 Triple Quadrupole LC/MS system (Agilent Technologies Inc., Santa Clara, CA, USA). The HPLC system was equipped with an ODS C18 analytical column (4.6 × 250 mm) with the detection wavelength of 258 nm. The column temperature was 25 °C. The mobile phase consisted of ddH_2_O (A) and acetonitrile (B) with a ratio of 50:50 (*v*/*v*) at a flow rate of 0.3 mL/min was used. Analytical identification was performed using multiple reactions monitoring (MRM) and electrospray ionisation (ESI) in negative mode. The operation conditions were as follows: capillary 4000 V, nebulizer 35 psi (N_2_), dry gas flow rate 10 L/min at 350 °C. Agilent MassHunter Workstation Data acquisition software (Agilent Technologies Inc.: Santa Clara, CA, USA) was used for data acquisition and processing.

### 3.6. Antioxidant Activity of Purified Mangiferin

The DPPH^•^ scavenging activity was measured according to Brand-williams *et al*. [27] with modifications. The reaction for scavenging DPPH^•^ radicals was carried out by adding 0.1 mL sample to 3.9 mL 60 μmol/L DPPH^•^ solution at room temperature. After 30 min, absorbance at 515 nm before (*A*_0_) and after (*A*_1_) the reaction was recorded by a spectrophotometer (UV-2550, Shimadzu, Tokyo, Japan). The percentage of the decrease in absorbance over the initial absorbance [(*A*_0_ − *A*_1_)/*A*_0_] was used to calculate the radical scavenging activity.

The ferric reducing ability of plasma (FRAP) was measured according to Benzie and Strain [28] with modifications. The fresh working solution was prepared by mixing 100 mL 300 mmol/L acetate buffer (pH 3.6), 10 mL 10 mmol/L TPTZ solution in 40 mmol/L HCl, and 10 mL 20 mmol/L FeCl_3_ solution. The reaction was carried out by adding 0.1 mL sample to 0.9 mL of the FRAP solution for 10 min at 37 °C, and absorbance at 593 nm was recorded using a spectrophotometer.

For both assays, Vc and Trolox were assayed at the same time as controls.

### 3.7. HUVEC Protective Activity Assay

HUVEC were isolated and cultured according to Yang *et al*. [29]. Briefly, cells were cultured in plastic tissue culture flasks grown to confluence in DMEM medium containing 10% fetal bovine serum supplemented with 2 mmol/L l-glutamine, 100 IU/mL penicillin, 100 μg/mL streptomycin. HUVEC at log phase were used for the SRB assay. Cells were seeded in 96-well microtiter plates (3000 cells per well) and incubated with 100 μL medium per well and H_2_O_2_ of different concentrations for 24 h. Cells were then treated with different concentrations of mangiferin (10 μg/mL or 20 μg/mL), and incubated at 37 °C in 5% CO_2_ atmosphere for 24 h. Afterwards, 100 μL of SRB solution (4 mg/mL) was added to each well to stain for 20 min at room temperature, and then washed by 1% acetic acid. After drying, 100 μL of Tris solution was added to each well to dissolve SRB. The optical density (OD) was read at 515 nm using a micro plate reader (Thermo Electron Corporation, Waltham, MA, USA). Inhibitory rate (%) = (OD_control_ − OD_treatment_)/OD_control_ × 100%.

### 3.8. Statistic Analysis

Experiments were performed in triplicate and data were expressed as the mean ± standard deviation. Student’s *t*-test was analyzed for data in Figure 6 and *p* < 0.05 was defined as significant.

## 4. Conclusions

Mangiferin in mango fruit was characterized by quantification, purification and biological evaluation in the present study. Mango peel and seed kernel were found to be good sources of mangiferin and peel of LPM fruit contains the highest mangiferin content among 11 Chinese cultivars tested. Purified mangiferin was identified by HPLC and LC-MS, and it showed high DPPH^•^ free-radical scavenging capacities and reducing power by FRAP. In addition, mangiferin showed significant protective activity in HUVEC under H_2_O_2_-induced stress, thus having potential in the prevention of oxidative stress-associated diseases. The established efficient purification method by combination of macroporous HPD100 resin chromatography and optimized HSCCC system is important for further comprehensive utilization of mango processing byproducts and extensive characterization of the pharmaceutical utilization of mangiferin.

## Figures and Tables

**Table 1:** Mangiferin content in different fruit tissues of 11 Chinese mango cultivars.

Cultivars	Mangiferin content (mg/g DW)		

	Peel	Pulp	Seed kernel
GF	0.23 ± 0.03	N.D.	0.38 ± 0.02
GR-265	3.76 ± 0.63	0.004 ± 0.001	0.63 ± 0.06
JHM	0.16 ± 0.05	N.D.	0.89 ± 0.05
LPM	7.49 ± 0.14	0.012 ± 0.003	0.67 ± 0.02
LXM	3.93 ± 1.48	N.D.	0.33 ± 0.03
MQS	1.91 ± 0.33	0.20 ± 0.07	0.14 ± 0.01
SLLK811	0.52 ± 0.09	N.D.	0.50 ± 0.05
TN-1	0.04 ± 0.01	N.D.	0.62 ± 0.02
XH-2	0.67 ± 0.36	0.008 ± 0.002	1.04 ± 0.14
YX-1	0.13 ± 0.10	N.D.	0.21 ± 0.005
ZHM	7.34 ± 0.13	0.002 ± 0.0002	2.43 ± 0.10

All data are presented as mean ± SD (*n*=3) on a dry weight (DW) basis. N.D.: not detected.

**Table 2:** Partition coefficient (*K* value) of mangiferin in different solvent systems.

Solvent system (*v*/*v*)	Ratio	*K*
*n*-Butanol–1% acetic acid	1:1	6.37
*n*-Butanol–methyl alcohol–1%acetic acid	1:0.1:1	2.56
*n*-Butanol–methyl alcohol–1%acetic acid	1:0.2:1	3.70
Ethyl acetate–water	1:1	<0.01
Ethyl acetate–methyl alcohol–water	1:0.1:1	<0.01
Ethyl acetate–methyl alcohol–water	1:0.3:1	<0.01
Ethyl acetate–*n*-butanol–water	4:1:5	1.76

**Table 3:** The purities and recoveries of mangiferin in the two-step purification procedure.

Purification step	Purity (%)	Recovery (%)	Yield (mg)
Crude extract	0.75	/	/
HPD100 resin chromatography	37.80	85.15	337.9 a
HSCCC	99.13	77.94	74.3 b

a The amount of sample was obtained from 20 g raw material by the HPD100 resin chromatography;

b The amount of compound was obtained from 250 mg resin-refined sample by HSCCC purification.
